# Utilization of Carbon Nanospheres in Photocatalyst Production: From Composites to Highly Active Hollow Structures

**DOI:** 10.3390/ma12162537

**Published:** 2019-08-09

**Authors:** Tamás Gyulavári, Gábor Veréb, Zsolt Pap, Balázs Réti, Kornelia Baan, Milica Todea, Klára Magyari, Imre Miklós Szilágyi, Klara Hernadi

**Affiliations:** 1 Research Group of Environmental Chemistry, Institute of Chemistry, University of Szeged, H-6720 Szeged, Tisza Lajos krt. 103, Hungary; 2 Department of Applied and Environmental Chemistry, University of Szeged, H-6720 Szeged, Rerrich tér 1, Hungary; 3 Institute of Process Engineering, Faculty of Engineering, University of Szeged, H-6725 Szeged, Moszkvai krt. 9, Hungary; 4 Nanostructured Materials and Bio-Nano-Interfaces Center, Interdisciplinary Research Institute on Bio-Nano-Sciences, Babes-Bolyai University, RO–400271 Cluj-Napoca, Treboniu Laurian 42, Romania; 5 Institute of Environmental Science and Technology, University of Szeged, H-6720, Szeged, Tisza Lajos krt. 103, Hungary; 6 Department of Molecular Sciences, Faculty of Medicine, Iuliu Haţieganu University of Medicine and Pharmacy, RO–400012 Cluj-Napoca, Romania; 7 Department of Inorganic and Analytical Chemistry, Budapest University of Technology and Economics, H-1111 Budapest, Hungary

**Keywords:** titanium dioxide, carbon spheres, hollow structures, composite, visible light, photocatalysis, phenol

## Abstract

Titanium dioxide–carbon sphere (TiO_2_–CS) composites were constructed via using prefabricated carbon spheres as templates. By the removal of template from the TiO_2_–CS, TiO_2_ hollow structures (HS) were synthesized. The CS templates were prepared by the hydrothermal treatment of ordinary table sugar (sucrose). TiO_2_–HSs were obtained by removing CSs with calcination. Our own sensitized TiO_2_ was used for coating the CSs. The structure of the CSs, TiO_2_–CS composites, and TiO_2_–HSs were characterized by scanning electron microscopy (SEM), infrared spectroscopy (IR), X-ray diffraction (XRD), X-ray photoelectron spectroscopy (XPS), and diffuse reflectance spectroscopy (DRS). The effect of various synthesis parameters (purification method of CSs, precursor quantity, and applied furnace) on the morphology was investigated. The photocatalytic activity was investigated by phenol model pollutant degradation under visible light irradiation (λ > 400 nm). It was established that the composite samples possess lower crystallinity and photocatalytic activity compared to TiO_2_ hollow structures. Based on XPS measurements, the carbon content on the surface of the TiO_2_–HS exerts an adverse effect on the photocatalytic performance. The synthesis parameters were optimized and the TiO_2_–HS specimen having the best absolute and surface normalized photocatalytic efficiency was identified. The superior properties were explained in terms of its unique morphology and surface properties. The stability of this TiO_2_–HS was investigated via XRD and SEM measurements after three consecutive phenol degradation tests, and it was found to be highly stable as it entirely retained its crystal phase composition, morphology and photocatalytic activity.

## 1. Introduction

TiO_2_ nanoparticles in the excited state are capable of generating free radicals resulting in photoinduced reactions like disinfection [1,2,3,4,5], degradation of various organic pollutants [6,7,8,9], and production of H_2_, as an alternative green energy source [8,10,11,12]. Despite the well-known advantages of TiO_2_ (it is cheap, chemically stable, and available in large quantities in pure form), it has relatively wide band gap (3.02 eV for rutile and 3.20 eV for anatase [13]). This feature limits the scope of its practical applications as it can only be excited efficiently by UV photons. Over the last three decades, a large number of publications were dedicated to the improvement of the photocatalytic activity and the excitability of TiO_2_. These included doping with various non-metals [2,6,10,14], modifying with noble metals [15,16,17], sensitizing with dyes [18,19], synthesizing TiO_2_ with various morphologies [11,20], and coupling TiO_2_ with other semiconductors [21,22,23]. A promising direction is the use of different forms of carbon, e.g., carbon nanotubes [2,24,25,26], graphite oxide [27], activated carbon [7], graphene [28], and graphene oxide [1]. Carbon materials have the potential of improving the photocatalytic activity of TiO_2_ by (1) narrowing the band gap of the semiconductor, (2) decreasing the recombination rate of photogenerated charge carriers, (3) providing more active reaction/adsorption centers in greater amounts and higher surface area, (4) acting as a photosensitizer for the photocatalytic reactions, and (5) prolonging the lifespan of charge carriers [27,29,30]. There are numerous examples described in the literature where most of these advantageous effects were observed when amorphous (that is, not crystalline) carbon was used [31,32,33].

Recently, semiconductor-based hollow structures have been extensively investigated due to their exceptional optical, optoelectronic, magnetic, electrical, thermal, and chemical properties [34,35,36,37]. Multiple reflections within the hollow cavity may lead to more efficient light harvesting resulting in the production of more photogenerated charge carriers, thus increasing the photocatalytic efficiency of the semiconductor. For the preparation of spherical hollow structures the use of various methods (e.g., hydrothermal, sonochemical, and template-based) [38] have been reported. One of the most appropriate template materials are carbon spheres, due to their environmentally friendly nature and the high hydrophilicity due to the polar –OH and –C=O surface groups [39]. Additionally, the properties of these spheres are finely tunable, including diameter, surface area, accretion and carbon layer arrangement [40,41]. The beneficial effects of the unique morphology on the photocatalytic activity have been reported in numerous publications [38,42].

In the current paper, CSs were prepared by a facile, hydrothermal method from ordinary table sugar and used as templates to synthesize TiO_2_–CS composites and TiO_2_–HS by template removal method. For the TiO_2_ coating our own synthesis method (from a previous publication [43]) was used, which resulted in highly visible light-active TiO_2_ (denoted as ‘Rutile-H2’). To the best of our knowledge, coating carbon spheres with TiO_2_ rather than using titanium dioxide precursors (i.e., using existing synthesis method to form TiO_2_ coating) has not been reported so far. Moreover, publications about visible light-active titania hollow structures are scarce, so a further aim of this paper was to give information about beneficial synthesis parameters in relation with visible light-active hollow structural titanium dioxides. To this, an attempt was made to further increase the photocatalytic activity of the Rutile-H2 sample and to describe in detail the differences of its structure and photocatalytic activity before (composite material) and after (hollow structure) removing the CS template, in the case of visible light irradiation.

## 2. Materials and Methods

### 2.1. Materials

To prepare the TiO_2_–CS composites and TiO_2_ hollow structures, Ti(IV) butoxide (Sigma-Aldrich, Schnelldorf, Germany; reagent grade; 97%), HCl (VWR Chemicals, Debrecen, Hungary; 37%), NaOH (Molar Chemicals, Halásztelek, Hungary; a.r.; 50%) H_2_O_2_ (Sigma-Aldrich, Schnelldorf, Germany; 30%), and ultrapure water (Millipore Milli-Q, Budapest, Hungary) were used. Carbon sphere templates were synthesized by using ordinary table sugar (sucrose, Magyar Cukor Zrt., Koronás^TM^, Kaposvár, Hungary) as carbon source, acetone (Molar Chemicals; 99.96%), and Milli-Q water for their purification. As model pollutant phenol (Spektrum 3D; analytical grade) was used to examine the photocatalytic activities. Our own ‘Rutile-H2’ TiO_2_ (a nonhollow structural, rutile TiO_2_ with improved visible light excitability, published in our previous work [43]) and ‘Rutile-H2_calc’ (a TiO_2_ synthesized by the calcination of Rutile-H2 the same way as the TiO_2_ hollow structures were synthesized, only without the addition of CS templates) were used as references together with commercial Evonik Aeroxide P25.

### 2.2. Preparation of the Carbon Sphere Templates

The synthesis method of carbon sphere templates (CSs) was based on our recent publication [41]. In a Teflon-lined stainless steel autoclave (V_total_ = 623 mL), 180.7 mL 0.15 M sucrose solution was prepared and the pH was set to 12 using 2 M sodium hydroxide (V_fill_/V_total_ = 29%). Then, the hydrothermal treatment of CSs was carried out by placing the autoclave in a drying oven at 180 °C for 12 h. Subsequently, the brownish-black product was collected as soon as the autoclave cooled down to room temperature. To remove the residual organic contaminants formed during the hydrothermal synthesis, two different purification methods were applied and compared: membrane filtration and centrifugation. For the membrane filtration 250 mL of the CS suspension was membrane filtered through a polyvinylidene fluoride (PTFE) ultrafilter membrane in a batch-stirred membrane reactor (Millipore, XFUF07601, Burlington, MA, USA) under vigorous magnetic stirring until the volume reduction ratio (VRR) was 5 (200 mL permeate and 50 mL concentrate were produced). Then, the concentrate was purified by various cycles of acetone or Milli-Q water addition, followed by repeated filtrations. One cycle meant the addition of 200 mL acetone or Milli-Q water and the subsequent filtration, until reaching the desired VRR = 5 value. The lowest degree of purification was carried out by the utilization of 1 acetone and 1 Milli-Q cycle, a medium purification by 3 acetone and 1 Milli-Q cycles, and, meanwhile, the most purified CS was prepared by 5 acetone and 2 Milli-Q water cycles, respectively. Total amounts of the employed acetone and Milli-Q water were 100–500 mL/g CS and 100–200 mL/g CS, respectively. At the second part of the research, centrifugation was used for the purification of CSs, which proved to be a more feasible method. CSs were washed in three cycles using solely acetone. The rate of centrifugation was 13,400 rpm and the total amount of acetone was 80 mL/g CS. At the end of both purification methods, the solid product was dried in air at 40 °C and was ground in agate mortar.

### 2.3. Preparation of the TiO_2_–CS Composites and TiO_2_ Hollow Structures

For the synthesis of the TiO_2_ coating our own synthesis method (described in detail in our recent publication [43]) was applied (resulting the ‘Rutile-H2’ TiO_2_), as follows. For a typical synthesis, 3.5 mL Milli-Q water was poured into a beaker and 1.3 mL hydrogen peroxide, and 1.58 mL hydrochloric acid was added to it under vigorous magnetic stirring. Then, to the previously prepared solution 2.14 mL Ti(O-nBu)_4_ was added dropwise at a constant rate of 1 mL∙min^−1^. Subsequently, after waiting 60 min to ensure the phase separation of the organic and inorganic phase of the titanium dioxide precursor, the bottom, dark orange colored inorganic phase containing the peroxo-titanium complex was isolated using a separatory funnel. In the next step, the coating process took place by adding 1.9 g of the previously prepared CSs to the titania containing inorganic phase, which was then sonicated for 10 min. The as-prepared suspension was aged for 48 h at 55 °C to initiate the crystallization process, resulting in the TiO_2_–CS composites. Afterwards, CSs were eliminated by calcination using either muffle furnace (Nabertherm B 180 type) without additional air supply or a tube furnace (Thermolyne 21100 type) under continuous air supply (0.5 dm^3^∙min^−1^) with a heating rate of 5 °C∙min^−1^ for 4 h at 400 °C resulting the TiO_2_–HSs (based on the TG-DTG measurements of our previous publication applying 400 °C was sufficient to remove the CS templates [41]). The names of the TiO_2_ samples are as follows; ‘H2_HS_X’, where H2 refers to our previous ‘Rutile-H2’ TiO_2_ whose synthesis method was used for the coating process, HS refers to hollow structures, and X is the number indicating the synthesis conditions. The applied CSs, the name of the TiO_2_ samples and the various synthesis conditions were summarized in Table 1.

### 2.4. Characterization Methods and Instrumentation

X-ray diffractograms were acquired by using a Rigaku diffractometer. The settings for the measurements were 30 mA, 40 kV, λ Cu Kα = 0.15406 nm, and the 20–40 (2θ°) interval was recorded. The Scherrer equation was applied for the evaluation of mean primary crystallite sizes. For the estimation of anatase and rutile weight fractions the peak areas at 25.3 (2θ°) and 27.5 (2θ°) were utilized, respectively, using the following equation [44,45].

$$W_{R}=\frac{A_{R}}{0.884\times A_{A}+A_{R}}$$

where W_R_ is the ratio of rutile crystal phase and A_A_ and A_R_ are the peak areas of anatase and rutile, respectively.

A Hitachi S-4700 Type II scanning electron microscope (SEM, Tokyo, Japan) was used to determine the samples’ particle sizes. For the production and acceleration of electron beam a cold field emission gun and 10 kV acceleration voltage was applied, respectively. The morphology was observed by collecting the secondary electrons with an Everhart–Thornley detector. The as-recorded SEM micrographs were used to examine the diameters of the hollow structures.

To obtain the diffuse reflectance spectra (DRS), a Jasco-V650 spectrophotometer (JASCO Deutschland GmbH, Pfungstadt, Germany) was used, which was equipped with an ILV-724-type integration sphere. The spectra were recorded in the λ = 220 to 800 nm region.

FT-IR measurements were conducted on a Jasco 6000 spectrometer. The parameters for the process were spectral resolution 4 cm^−1^, 400–4000 cm^−1^ range. For the evaluation of TiO_2_ samples, they were added to KBr powder, from which pellets were produced.

The BET method was used to calculate the specific surface areas of the titanium dioxide samples, for which a BELCAT-A device was used to record the isotherms at 77 K via N_2_ adsorption.

For the X-ray photoelectron spectroscopy (XPS) measurements a Specs Phoibos 150 MCD type device was used. The apparatus included the following parts: a hemispherical analyzer, a monochromatic Al-Kα source (1486.6 eV) at 14 kV and 20 mA, and a charge neutralization device. For the measurements the samples were installed on double-sided carbon tapes. The applied settings during the process were as follows: X-ray source, 200 W, and the pressure in the analysis varied between 10^−9^–10^−10^ mbar. The binding energy scale was charge referenced to the C1s at 284.6 eV. Analyzer pass energy of 20 eV in steps of 0.05 eV was applied to obtain high resolution Ti2p and O1s spectra. CasaXPS software was used for the evaluation of the data.

### 2.5. Evaluation of the Photocatalytic Efficiencies

**Figure 1:**
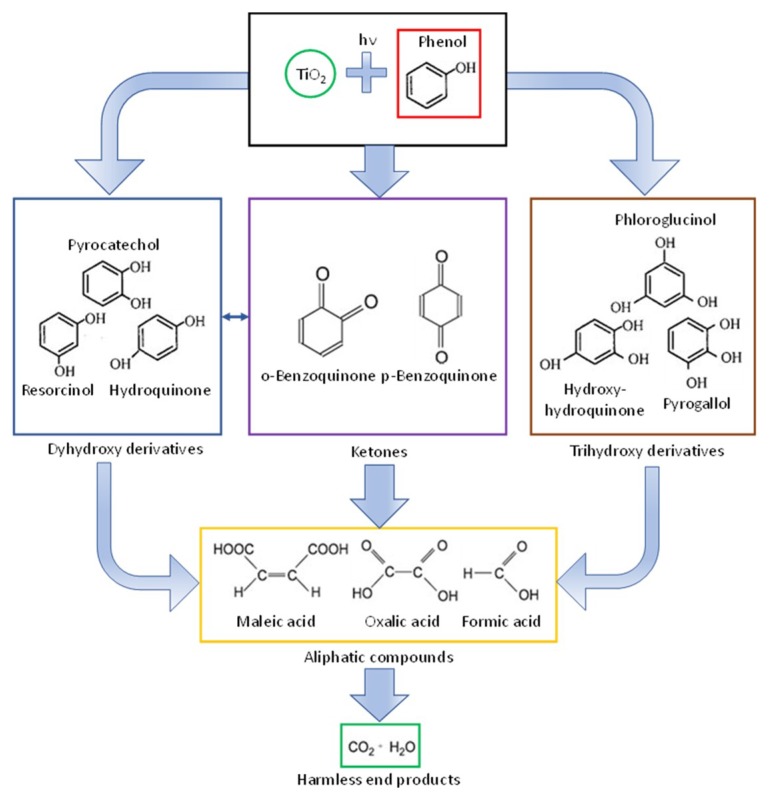
Possible intermediates of phenol during its photocatalytic oxidation [46,47,48].

Photocatalytic performance of the TiO_2_–CS composites and hollow structures was investigated by the photocatalytic decomposition of phenol (c_phenol_ = 0.1 mM) under visible light irradiation (λ > 400 nm). For the excitation of the photocatalysts, 4 conventional energy saving lamps (Düwi 25920/R7S-24W) were used, which surrounded a double-walled glass vessel containing the samples. During the entire procedure a constant temperature of 25 °C was set, and air was continuously supplied into the photoreactor via a glass tube to maintain the dissolved oxygen level. In the thermostating jacket of the glass vessel 1 M NaNO_2_ solution was circulated in order to cut-off UV photons (λ < 400 nm). The emission spectrum of the lamp can be seen in Figure S1. The suspensions of the photocatalysts containing the model contaminant phenol were left to be stirred in the dark for 30 min to reach adsorption/desorption equilibrium. The changes in the phenol concentration were followed by a Hitachi high performance liquid chromatography (HPLC) system equipped with a Lichrospher RP 18 column. As eluent, 50–50% methanol/water mixture was applied, and the detection wavelength was 210 nm. The possible mechanism of the photocatalytic oxidation of phenol has been extensively investigated in the literature [46,47,48] and based on the published results a figure representing the possible reactions and byproducts of phenol was constructed (Figure 1), which was used to investigate the intermediates formed during the degradation process.

## 3. Results and Discussion

### 3.1. Characterization of the Carbon Sphere Templates

**Figure 2:**
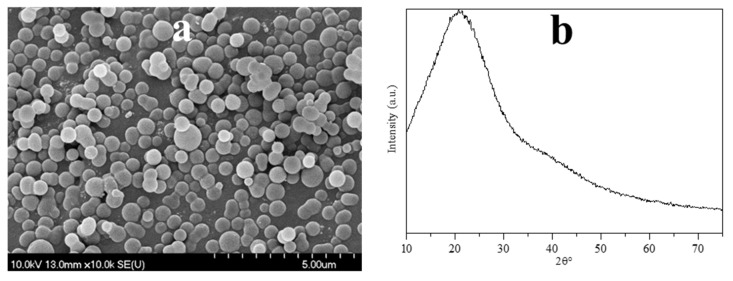
Typical SEM micrograph (**a**) and XRD diffractogram (**b**) of the obtained carbon spheres.

**Figure 3:**
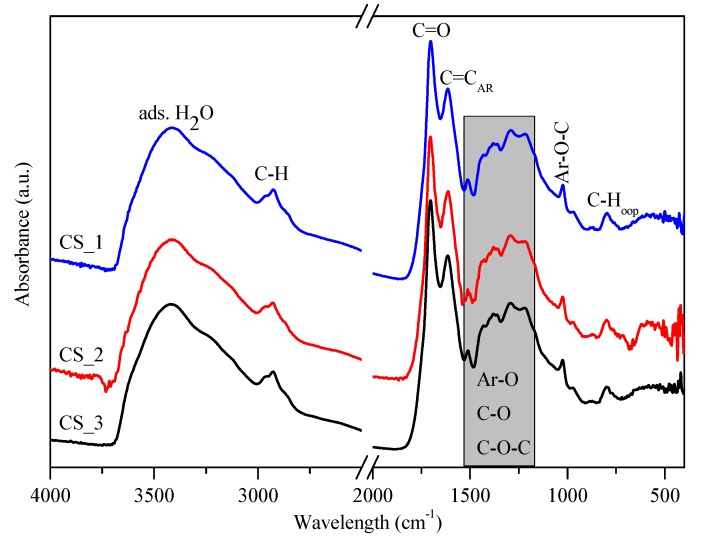
IR spectra of carbon spheres purified with increasing amounts of acetone (CS_1 < CS_2 < CS_3, respectively) by membrane filtration.

According to X. Sun and Y. Li, during the hydrothermal synthesis of carbon spheres the following steps occur (in accordance with the LaMer model [49]); (i) after reaching the necessary concentration and temperature of the solution, aromatic compounds and oligosaccharides form (indicated by the increasing viscosity and the orange/red color of the solution), then polymerization occurs; (ii) the solution reaches supersaturation resulting in nucleation followed by the cross-linking of linear/branch-like oligosaccharides and other macromolecules; and (iii) the as-formed nuclei grow uniformly and isotropically by the diffusion of solutes reaching its final size [50]. To remove the previously mentioned organic residues, two different methods were applied: membrane filtration and centrifugation. In our previous publication the carbon spheres were purified with vacuum filtration and characterized in detail [41]; however, in this work, new purification methods were applied in order to develop a more suitable method providing better yield, since vacuum filtration had very low flux (resulting in low CS yield) due to the fouling of the filter; meanwhile, membrane filtration enabled magnetic stirring to be applied during the filtration. Based on the SEM and XRD analysis, no differences were observed between the variously purified CSs, thus one typical SEM micrograph and XRD diffractogram were represented in Figure 2. The SEM micrographs showed well-defined sphere morphology with a mean diameter of 458 nm ± 75 nm. Based on the XRD analysis, one broad peak was observed at ~22 (2θ°), which was attributed to amorphous carbon, and no other reflections were detected, as expected [41]. The correlation between carbon sphere purity and the quality of the resulting hollow structures was investigated by using CSs purified via membrane filtration with different amounts of acetone, which were characterized further by FT-IR measurements (Figure 3). Additionally, the carbonization of sucrose solution during the hydrothermal treatment was also examined. Absorption bands at 3420, 2920, 1700, and 1615 cm^−1^ can be attributed to physisorbed water, C–H stretching vibration, C=O stretching vibration, and C=C vibration in the aromatic ring, respectively [51]. The signals in the spectral range of 1100 to 1400 cm^−1^ correspond to C–OH stretching vibration and OH bending vibration, indicating the presence of hydroxyl groups in large quantities [50,52]. The FT-IR spectra indicated no significant difference between the quality of the carbon spheres’ surface and purity, from which it can be presumed, that by the utilization of these CSs no considerable difference should be observable in the properties of the resulting HSs.

### 3.2. Comparison of the TiO_2_–CS Composite and TiO_2_ Hollow Structure

TiO_2_–CS composite (denoted as ‘H2_CS_0’) was synthesized by coating CS purified by membrane filtration with our previous ‘Rutile-H2’ titanium dioxide (via the method described in Section 2.3), and was compared to the TiO_2_ hollow structure (denoted as ‘H2_HS_0’), which was obtained by the calcination of the composite in muffle furnace. The as-prepared composite and hollow structure were characterized by XRD (Figure S2), and it was found that both contained anatase phase (72% and 62% and D = 9.7 nm, 14.2 nm for H2_CS_0 and H2_HS_0, respectively) to a larger extent, compared to rutile phase (28%, 38% and D = 16.4 nm, 17.9 nm, for H2_CS_0 and H2_HS_0, respectively). The main difference in the diffraction patterns of the 2 samples was the level of crystallinity (the intensity of the diffractions of H2_HS_0 was much higher), which can be explained by the result of calcination (as well as the increase in crystallite sizes). This result is in good accordance with our previous observations that applying amorphous carbon results in the coating being amorphous and, similarly, that using crystalline carbon template results in crystalline coating [53]. Afterwards, these samples were characterized by IR measurements (Figure S3) and as expected, all of the peaks observed earlier in the previous section in case of CSs, also appeared in case of the composite sample. Additionally, a less intense peak at 1029 cm^−1^ was detected, which can be attributed to residues of disaccharides [54,55]. The broad absorption band between 400–600 cm^−1^ can be assigned to the transverse optical vibration of Ti–O bonds [56], proving the presence of TiO_2_ coating layer. Most importantly, these measurements were followed by the evaluation of photocatalytic activity (Figure S4), and it was found, that H2_CS_0 possessed less than half of the photocatalytic efficiency of the H2_HS_0 sample. To sum up, it was observed, that compared to Rutile-H2, the presence of carbon spheres significantly decreased the crystallinity and photocatalytic activity of the sample. Concurrently, compared to H2_CS_0 composite sample, after calcination, the H2_HS_0 sample possessed higher crystallinity as expected and also higher photocatalytic activity, which can be attributed to the resultant effect of higher crystallinity and presumably, the increased light-harvesting capabilities due to the unique morphology. Based on that discovery, the composite samples (and H2_HS_0 TiO_2_) were not investigated and discussed further in this paper, and more emphasis were put on the characterization of hollow structures, since despite the increased photocatalytic activity of the hollow structural TiO_2_, (in comparison with the composite material) the photocatalytic activity of Rutil-H2 still has not been exceeded.

**Figure 4:**
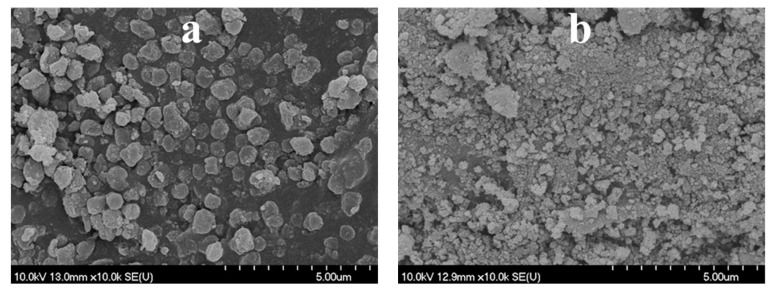
SEM micrographs of the H2_HS_1 sample before (**a**) and after (**b**) calcination.

**Figure 5:**
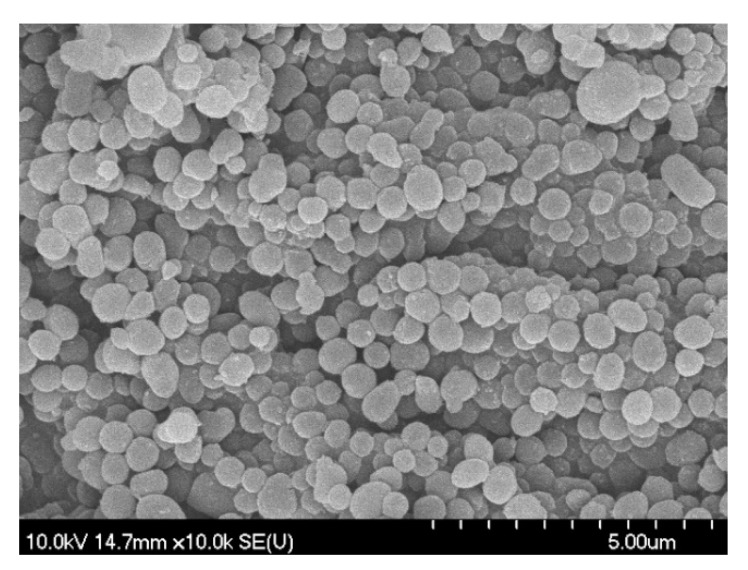
Typical SEM micrograph of samples H2_HS_2-4 before calcination.

As the next step, our aim was to investigate if by the application of variously purified carbon spheres and different synthesis parameters the beneficial properties of the unique morphology can be utilized, thus surpassing the photocatalytic activity of the reference Rutile-H2 TiO_2_. By the utilization of the various as-prepared carbon spheres, TiO_2_–CS composites were synthesized, then, by their subsequent calcination, the TiO_2_ hollow structures were obtained, which will be discussed in detail. The applied CSs, the name of the TiO_2_ samples and the various synthesis conditions were summarized in Table 1. For the first synthesis of composite and HS, membrane filtered CS was applied, and the applied quantity of the precursor was three times higher compared to the one written in Section 2.3. After performing the coating and subsequent SEM measurement, it was found that the quantity of TiO_2_ precursor was too high, which resulted in the formation of irregular TiO_2_–CS composite (in terms of shape) and TiO_2_ nanoparticle aggregates (Figure 4a). Accordingly, after calcination (sample denoted as ‘H2_HS_1’) no regular HSs were observed (Figure 4b) therefore the quantity of the TiO_2_ precursor was reduced to one third (as the volumes are described in Section 2.3), which resulted in successful coating of the CSs (Figure 5).

**Figure 6:**
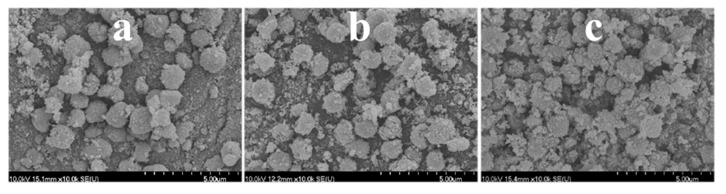
SEM micrographs of the H2_HS_2 (**a**), H2_HS_3 (**b**), and H2_HS_4 (**c**) samples (synthesized by the application of membrane filtered carbon spheres using increasing amounts of acetone/water, respectively) after calcination.

By the utilization of CSs purified by membrane filtration with increasing amount of acetone/water (denoted as CS_1, CS_2, and CS_3, purified by the application of 1, 3, and 5 acetone washing cycles and 1, 1, and 2 Milli-Q washing cycles, respectively), further TiO_2_ HSs were synthesized (denoted as ‘H2_HS_2-4’), which displayed the desired hollow structural morphology based on the SEM micrographs (Figure 6a–c); however, the morphology was still imperfect, as a significant amount of damaged structures were also observed.

**Figure 7:**
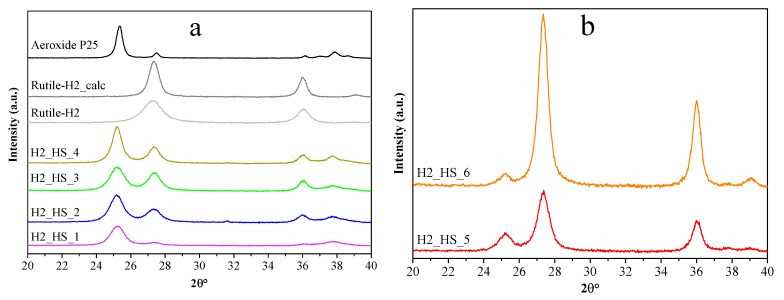
X-ray diffraction patterns of our own anatase phase and reference TiO_2_-s (**a**) and our own rutile phase TiO_2_-s (**b**).

The quality of HSs was mostly uniform, which is in good accordance with the IR results, since the features of the CSs’ surface were generally identical. At this point, the crystallinity of the as-prepared TiO_2_ HSs, and reference photocatalysts (Evonik Aeroxide P25, Rutile-H2, and Rutile-H2_calc) was determined by XRD measurements (Figure 7a). The crystal phase distributions and average primer particle sizes are presented in Table 2. Samples H2_HS_1-4 contained predominantly anatase (1 0 1) phase (main diffraction peak at 25.6 2θ°). The size of the primer crystallites, which were the building elements of the hollow structures, varied between 9.3 and 15.0 nm. Reference photocatalysts Rutile-H2 (D = 7.3 nm) and Rutile-H2_calc (D = 14.1 nm) contained only rutile (1 1 0) (main diffraction peak at 27.2 2θ°). The size increase (and concurrent considerable loss of specific surface area) of Rutile-H2_calc can be explained by the result of calcination. Commercial Aeroxide P25 contained 90 wt% anatase and 10 wt% rutile (D_anatase_ = 25.4 nm and D_rutile_ = 40 nm).

To sum up the results so far, the synthesis parameters applied in the case of sample H2_HS_1 were not adequate, but after the reduction of precursor quantity (samples H2_HS_2-4), the coating process was successful (in the case of all the subsequent samples). However, the morphology was still imperfect (because of the considerable number of damaged spheres formed during the calcination) and our samples contained mainly anatase phase which is not excitable by visible light. Also, membrane filtration as a purification method did not prove to be feasible and economical enough: until achieving VRR = 5 volume reduction ratio in every cycle, still high amount of residuals (~20% of the initial amount of the given cycle) can remain in the solution (and higher VRR would be very hard to achieve, due to the observed significant flux reduction, despite the intensive stirring). Three times repeated centrifugation can result in several orders of magnitude higher purification efficiency of the CSs. Additionally, in the publication of Mahyar et al. they showed, that media with different polarity (a series of primer alcohols) can set up the formation of different crystal phases [57]. Based on this, it can be assumed that by applying different solvents for washing the surface of the CSs, differences in the quality of the surface can be induced, influencing the forming crystal phase composition of the deposited TiO_2_. Since our goal was to synthesize visible light-active TiO_2_-s, to which rutile phase is more advantageous (as it is excitable by visible light), further on only acetone was used as purifier solvent, and the more feasible centrifugation was applied as the purification method of CSs (sample H2_HS_5).

**Figure 8:**
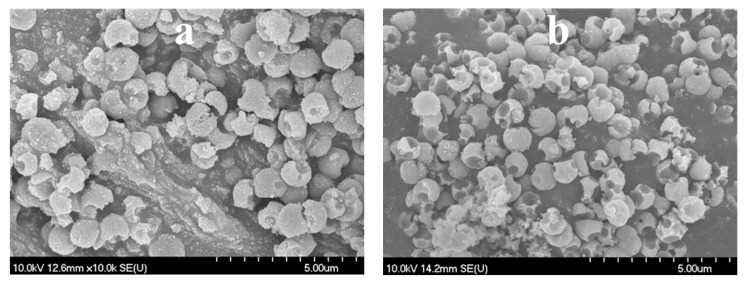
SEM micrographs of the H2_HS_5 (**a**) and H2_HS_6 (**b**) samples.

Moreover, since after the reduction of precursor quantity the coating process was always successful and the damaged structures were obtained only after calcination, another sample was synthesized where tube furnace with air supply was used for the elimination of carbon spheres (sample H2_HS_6), in contrast with using muffle furnace without additional air supply (samples H2_HS_1-5). Applying such synthesis parameters, samples H2_HS_5-6 both possessed perfect hollow structural morphology (Figure 8a,b) and were predominantly rutile phase (Figure 7b). Based on their greater rutile content and, more importantly, the perfect morphology, it is expected that these TiO_2_-s (H2_HS_5-6) should have increased photocatalytic efficiency under visible light irradiation compared to the other TiO_2_-s in the series (H2_HS_1-4), as rutile absorbs visible light (having a band gap of 3.02 eV [13]) and anatase does not, the hollow structure may enable better utilization of visible light, as this was already proved in the case of UV irradiation of TiO_2_ hollow structures [38,42]. To sum up, it was deduced, that the quality of HS morphology is largely dependent on the precursor quantity and the purification method of carbon spheres.

**Figure 9:**
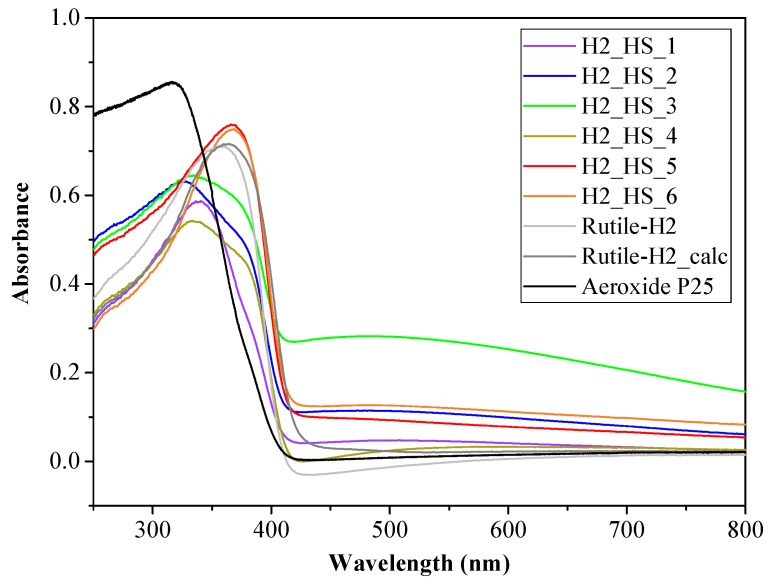
Diffuse reflectance (DR) spectra of the investigated TiO_2_-s.

**Figure 10:**
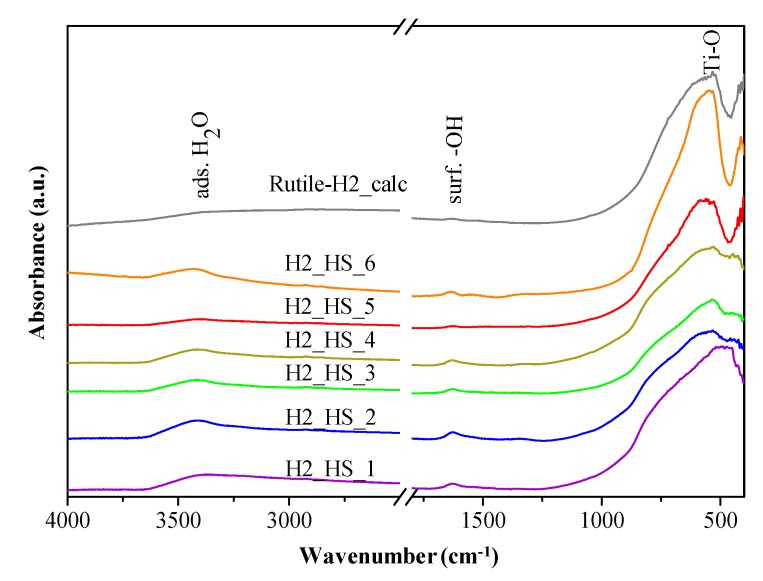
IR spectra of the investigated TiO_2_-s.

The light absorption of the TiO_2_-s was determined by diffuse reflectance spectroscopy (DRS). As it can be seen in Figure 9, the TiO_2_ HSs possess greater light absorption in the visible light region compared to reference Rutile-H2 and Aeroxide P25. The band gap energies determined from the derivative DR spectra were 3.14, 3.14, 3.15, 3.13, 3.13, and 3.13 eV for H2_HS_1-6 photocatalysts, and 3.11, 3.09, and 3.11 eV for Rutile-H2, Rutile-H2_calc, and Aeroxide P25, respectively. Based on the DRS results, samples H2_HS_1–6 possess very similar values, thus possible differences in their photocatalytic activity presumably will not be attributable to their band gaps. Surface properties of TiO_2_ HSs were characterized by FT-IR spectroscopy (Figure 10). In the FT-IR, a spectrum with a broad band centered at 3400 cm^−1^ with a sharp band at 1630 cm^−1^ was observed, which can be associated with the stretching and bending vibrations of the surface OH groups [58,59]; moreover, the bands around 480 and 540 cm^−1^ correspond to Ti–O bond [56]. The shape of these latter bands in this region varied in accordance with the different crystal phases; anatase had less defined Ti–O stretch band as it possesses less ordered structure (compared to rutile); consequently, in the case of mixed crystal phases, the infrared absorption bands also represented this transmission between the two crystal phases [60].

### 3.3. Evaluation of Photocatalytic Activity

**Figure 11:**
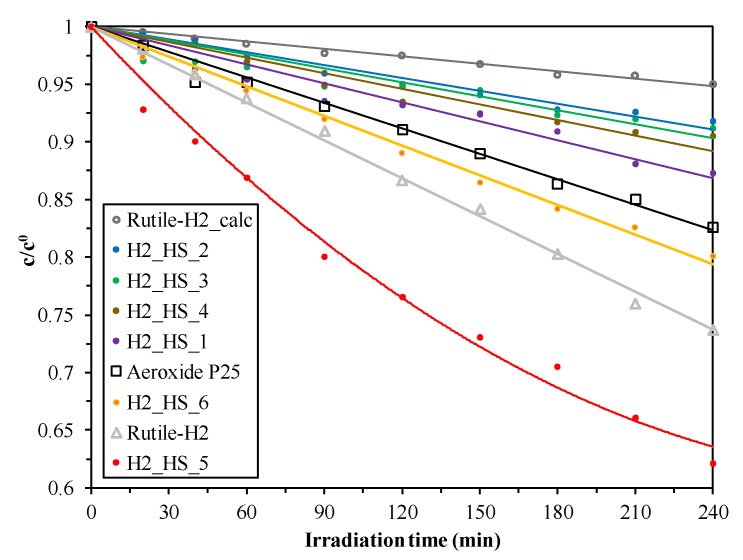
Photocatalytic decomposition of phenol solutions under visible light irradiation (c_phenol_ = 0.1 mM, c_TiO2_ =1.0 g∙L^−1^).

The photocatalytic degradation curves of phenol are presented in Figure 11. Samples with higher anatase content (H2_HS_1-4) possessed significantly lower photocatalytic efficiency compared to samples with higher rutile content (H2_HS_5-6), as was expected. In the case of the samples with higher photocatalytic activity, only the formation of resorcinol—a byproduct of phenol—was detected in measurable amount, and no significant differences in the amounts and evolution trends was observed. A typical chromatogram of the samples with high photocatalytic activity at the end of the measurements can be seen in Figure S5. H2_HS_5 TiO_2_ had outstanding photocatalytic performance, exceeding the photocatalytic activity of Aeroxide P25 and even our previous peroxo group containing Rutile-H2 TiO_2_ with high visible light activity, which could be explained by its rutile content and, more importantly, its perfect morphology, resulting in presumably the enhanced utilization of light source. Based on these results, it was deduced, that the activity gain caused by the hollow structural morphology significantly overcompensated the activity loss caused by the disappearance of peroxo groups during calcination. Moreover, H2_HS_5 TiO_2_ possessed not only the highest absolute photocatalytic activity, but the lowest specific surface area (out of the TiO_2_ series synthesized by the application of CSs), thus the highest surface normalized photocatalytic efficiency as well (8 times higher than the Rutile-H2; see in Table 2). It is important to highlight, that the photocatalytic activity of all samples possessing hollow structural morphology surpassed the photocatalytic efficiency of Rutile-H2_calc TiO_2_, which can be considered as the appropriate reference in terms of measuring the activity gain by the morphology, since it was synthesized similarly (including the calcination step), just without the application of carbon sphere templates. Lastly, it is important to mention that although samples H2_HS_5 and H2_HS_6 possessed very similar characteristic properties (high rutile content, similar morphology, band gap, crystallite size, and specific surface area), sample H2_HS_5 was still nearly twice as efficient compared to H2_HS_6, despite the latter having larger specific surface area and higher rutile content (the only difference was that in the case of H2_HS_5, carbon spheres were eliminated in muffle furnace without additional air supply, whereas in the case of H2_HS_6 tube furnace was used with constant air supply). In order to ascertain the cause of this result, these two samples (together with H2_HS_2 TiO_2_ as a reference sample with low photocatalytic activity) were characterized further by XPS measurements.

**Figure 12:**
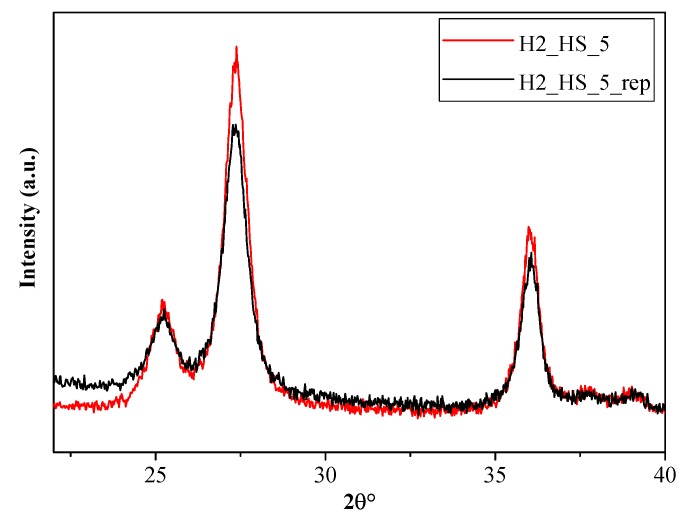
XRD diffractograms of H2_HS_5 before and after the 3 consecutive phenol degradation tests (H2_HS_5_rep).

**Figure 13:**
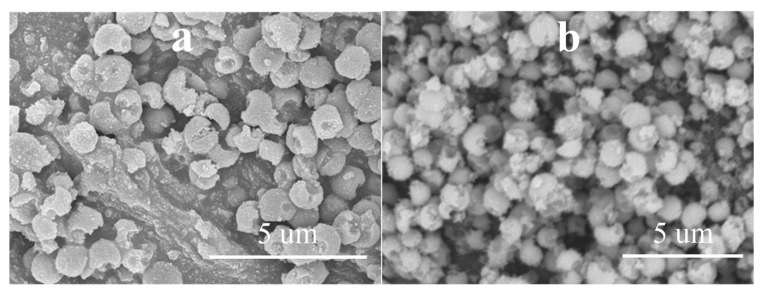
SEM micrographs of H2_HS_5 before (**a**) and after (**b**) the 3 consecutive phenol degradation tests (H2_HS_5_rep).

The stability of the sample H2_HS_5—possessing the highest photocatalytic activity—was investigated by XRD and SEM measurements after three consecutive phenol degradation tests, and this sample was named as ‘H2_HS_5_rep’. During the three runs the photocatalytic efficiency of H2_HS_5_rep did not decrease: the standard deviation was less than 2%. Based on the XRD diffractograms (Figure 12) sample H2_HS_5_rep entirely retained its initial crystal phase composition (80% rutile and 20% anatase) and its morphology as well (Figure 13), proving this catalyst to be particularly stable.

### 3.4. XPS Measurements

The studied materials were obtained by a synthesis process involving two crystallization steps: the first being the deposition of titania onto the surface of the carbon spheres, and its subsequent crystallization at 55 °C for 48 h, and the second was the removal of the carbon templates by calcination, while a recrystallization process occurred. The mentioned two synthesis steps are rather complex, suggesting that a more focused analysis was needed concerning the obtained nanostructures’ surface quality (the concertation of a specific element was given in at% in the case of all samples, while the distribution of specific oxidation states is given in % from the total amount of that specific element).

**Figure 14:**
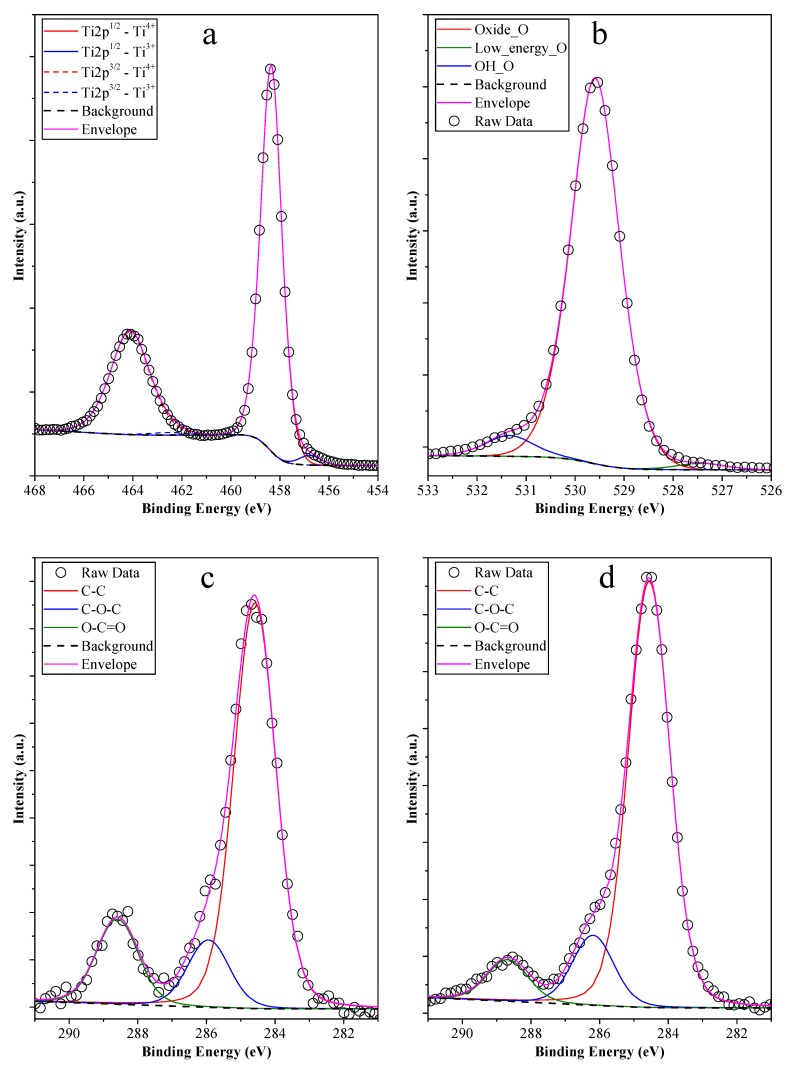

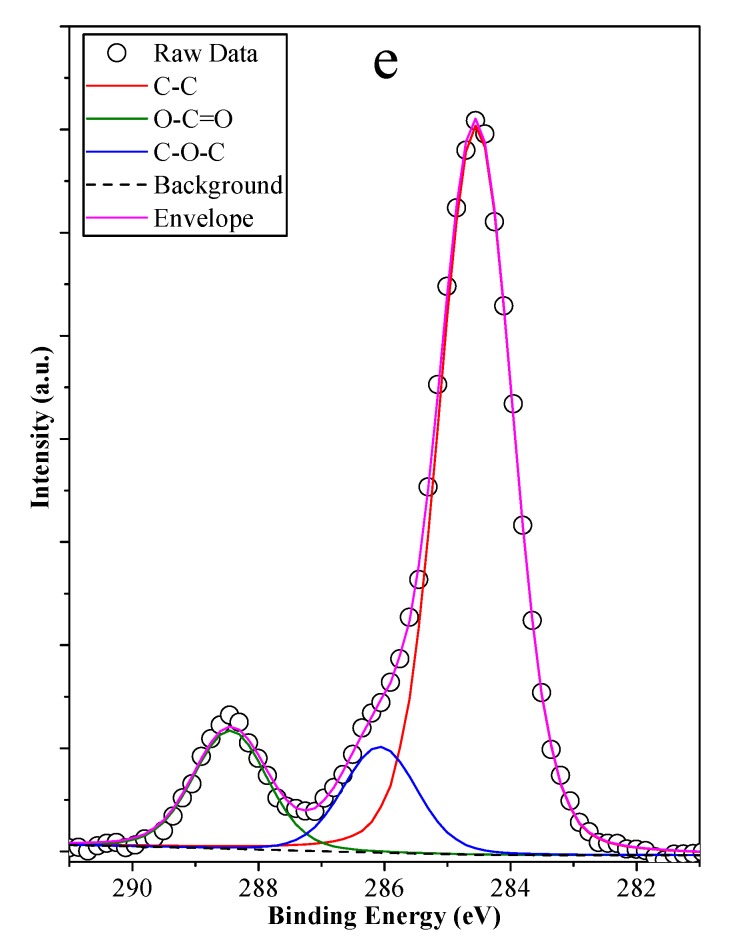
The core X-ray photoelectron spectroscopy (XPS) spectra of samples H2_HS_5 (**a**) Ti2p; (**b**) O1s; (**c**) C1s), H2_HS_2 (**d**) C1s, and H2_HS_6 (**e**) C1s pointing out the influence of the calcination and/or the quality of the carbon spheres on the surface chemistry of the titania hollow spheres.

For this, and to investigate the cause of the differences between the photocatalytic activities sample H2_HS_2, H2_HS_5, and H2_HS_6 were selected. In a lot of cases, the main reason behind the varying photoactivity of a catalyst is the presence of different induced defects (e.g., Ti^3+^ [61]), which may act as active centers on the surface of the catalyst, therefore, in the first instance Ti2p spectra (Figure 14a) of the chosen materials were investigated. It was found that Ti^4+^ (458.3 eV—2p^1/2^; 464.1 eV—2p^3/2^) was the main Ti species identified, accompanied by a small amount of Ti^3+^ centers (456.8 eV—2p^1/2^; 462.3 eV—2p^3/2^) which is an important species possibly contributing to achieve high photocatalytic activity [62]. The content of Ti^4+^ and Ti^3+^ was nearly the same in all samples (2.24–2.49% Ti^3+^ and 97.76–97.51% Ti^4+^), pointing out that possible differences in the photoactivity may lie elsewhere. The O1s spectra (Figure 14b) revealed that low binding energy oxygen (527.5 eV) was present, which is usually associated with Ti^3+^ centers [63], but the content of this species was nearly identical in the samples. Furthermore, surface OH associated oxygen (531.3 eV) and lattice oxygen (529.7 eV) were also detected, which are usual components in such samples. The first significant difference between the three samples was the higher content of OH_O (8.97% compared to 5.02% and 4.97%, respectively) in sample H2–HS_2, suggesting a higher water affinity (hydrophilicity) [63], a potential parameter that might define the photoactivity of this sample. The amounts of the different oxygen- and carbon-containing species are summarized in Table 3.

Nevertheless, all investigated samples are based on carbon nanospheres, which were eliminated by calcination. As the elimination of organic compounds and carbon can induce interesting structural changes [62,63,64], including surface sensitization, carbon deposits, and optical response changes; it was considered crucial to verify this issue. Residual carbon can also have various effects on the photocatalytic activity of titania [64], thus the results of XPS measurements were processed by taking this ascertainment into account.

C1s XPS spectra (Figure 14c–e) of the above-mentioned samples were analyzed, and three carbon species/bond types were identified: C–C at 284.5 eV, C–O–C at 286.0 eV, and O–C=O at 288.6 eV. As the hollow nanostructures were obtained by the removal of carbon, the presence of oxidized carbon was not a surprise. However, in sample H2_HS_5, the amount of oxidized carbon was higher (15.19%) than in H2_HS_2 (7.72%) and H2_HS_6 (12.38%). The more pronounced presence of oxidized carbon in the most efficient H2_HS_5 sample points out that the removability of carbon spheres was higher in this case, which is also reinforced by the lower content of total carbon (8.91 at%, compared to sample H2_HS_2—20.18 at% and H2_HS_6—30.72 at%). The presence of carbon at such concentration values is known, even without applying additional carbon source, as the authors have shown in a paper [63].

Comparing sample H2_HS_6 with H2_HS_5 the following observations were made.

- The total amount of carbon in sample H2_HS_6 (30.72 at%) was determined to be higher than that of sample H2_HS_5 (8.91 at%).
- Moreover, in sample H2_HS_5, the ratio of O–C=O bonds was higher (15.18% from the total amount of carbon species) than in the case of H2_HS_6 (12.38% from the total amount of carbon species), pointing out a more efficient carbon removal (oxidation) process in case of the latter sample.
- The fact that CS templates could be eliminated to a greater degree without additional air supply (muffle furnace) compared to their removability by providing additional air (tube furnace with constant air supply) was very surprising. The whole synthesis procedure and comparison was repeated three times, and the result was consistent in all cases. A plausible explanation for this could be that during the introduction of air, the partial pressure of oxygen increases within the system (tube furnace, in the case of sample H2_HS_6), facilitating its adsorption to the titania surface leading to the formation of an oxygen monolayer. This monolayer could act as a trap for the exiting CO_2_ hindering its transport through the titania shell. Since a TiO_2_ coating with a sensitized TiO_2_ layer containing peroxo groups (Ti–O–O–Ti) was used and the degradation of these peroxo groups might proceed more easily in the absence of additional oxygen (muffle furnace, in the case of sample H2_HS_5), the necessary oxygen for the elimination of CS templates (partly) could still be provided locally by the degradation of peroxo groups during the calcination process.
- The above listed observations emphasize the fact that, as the carbon was more efficiently eliminated from the samples (shown by the lower carbon content and higher amount of oxidized carbon species), the photocatalytic activity was higher (sample H2_HS_6 possessed only 52.6% of the photocatalytic activity of sample H2_HS_5), because in the case of the samples with lower photocatalytic activity, the surface available for the photocatalytic process could be partially blocked and therefore more hydrophobic as well. Simultaneously, if the ratio of the O–C=O in the surface deposited carbon was higher, again supports the observed activity increase, since polar functional groups facilitate the direct contact between water (the matrix of the photocatalytic process) and the surface of the photocatalyst.

## 4. Conclusions

The synthesis method of our own Rutile-H2 TiO_2_ was used to coat carbon sphere templates to obtain TiO_2_–CS composites, then, by their subsequent calcination, TiO_2_ hollow structures. A TiO_2_–CS composite and TiO_2_–HS sample were compared, and it was found that the addition of CS templates decreased the crystallinity and photocatalytic activity. A series of TiO_2__HSs was synthesized by applying different synthesis conditions (different purification methods of carbon spheres, different precursor quantity and furnace). Centrifugation was found to be the more feasible CS purification method. Applying muffle furnace (without additional air supply) was found to be more efficient to remove CS templates compared to tube furnace with air supply. TiO_2_ sample named as H2_HS_5 was synthesized by applying these conditions which resulted in TiO_2_ HS with excellent hollow structural morphology. H2_HS_5 TiO_2_ possessed both the best absolute and surface normalized photocatalytic activity among the investigated TiO_2_-s. On one hand, this was attributed to its increased light-harvesting capabilities (due to its unique morphology), and on the other, to the lowest carbon content on its surface and the higher ratio of the polar O–C=O functional groups, which presumably facilitate the direct contact between water (the matrix of the photocatalytic process) and the surface of the photocatalyst. The stability of this TiO_2_ proved to be remarkable as it retained its photocatalytic activity, crystal phase composition and morphology after three consecutive phenol degradation measurements.

## Figures and Tables

**Table 1:** Applied synthesis parameters during the preparation of TiO_2_-C composites and HSs (*: no HSs; **: moderate quality HSs; ***: excellent quality HSs).

CS Purification Method	Washing Cycles	TiO_2_	TiO_2_:CS Weight Ratio	Furnace	Quality of HSs
Membrane filtration	3	H2_HS_1	0.84	Muffle (without air supply)	*
Membrane filtration	1	H2_HS_2	0.26	Muffle (without air supply)	**
Membrane filtration	3	H2_HS_3	0.26	Muffle (without air supply)	**
Membrane filtration	5	H2_HS_4	0.26	Muffle (without air supply)	**
Centrifugation	-	H2_HS_5	0.26	Muffle (without air supply)	***
Centrifugation	-	H2_HS_6	0.26	Tube (with constant air supply)	***

**Table 2:** Phase composition, average primary particle sizes, band gaps, and specific surface areas of the investigated TiO_2_-s.

Titanium-dioxide	Phase Composition								
	Anatase		Rutile						
	wt%	Particle Size (nm)	wt%	Particle Size (nm)	Average Diameter (nm)	Specific Surface Area (m^−2^ g^−1^)	Band Gap (eV)	r_0,phenol_ (10^−10^ M s^−1^)	r_0,phenol_ (10^−12^ M m^−2^ s^−1^) Surface Normalized
H2_HS_1	87.72	9.7	12.28	10.7	-	89	3.14	8.1	9.1
H2_HS_2	66.42	10.3	33.58	9.7	1202	70	3.14	6.03	8.6
H2_HS_3	56.91	9.3	43.09	10.8	1137	65	3.15	5.26	8.1
H2_HS_4	64.1	15	35.9	12.9	1237	46	3.13	7.02	15.3
H2_HS_5	20.0	12.3	80.0	12.9	880	40	3.13	24.63	61.6
H2_HS_6	4.8	18	95.2	15.5	943	46	3.13	13.65	29.7
Rutile-H2	<1	-	>99	7.3	-	237	3.11	18.3	7.7
Rutile-H2_calc	<1	-	>99	14.1	-	39	3.09	3.4	8.8
Aeroxide P25	90	25.4	10	40	-	49	3.11	12.3	25.1

**Table 3:** The amounts of different oxygen- and carbon containing species in samples H2_HS_2, H2_HS_5, and H2_HS_6. * From the total amount of O; # From the total amount of C.

Species (at%)	Sample Name		
	H2_HS_2	H2_HS_5	H2_HS_6
OH_O*	8.97	5.02	4.97
Oxide_O*	89.58	93.41	93.55
Low_O*	1.45	1.56	1.48
C–O–C#	13.09	12.07	11.11
O-C=O (oxidized carbon)#	7.72	15.19	12.38
Total_C (at%)	20.18	8.91	30.72

## Supplementary Materials

The following are available online at https://www.mdpi.com/1996-1944/12/16/2537/s1; Figure S1: Emission spectrum of the visible light emitting lamps used for the photocatalytic activity measurements, Figure S2: X-ray diffraction patterns of H2_CS_0 and H2_HS_0 samples, Figure S3: IR spectra of the investigated H2_CS_0 and H2_HS_0 samples, Figure S4: Phenol degradation curves of the H2_CS_0, H2_HS_0 and reference Rutile-H2 samples, Figure S5: Chromatogram of the most efficient H2_HS_5 sample by the end of the photocatalytic oxidation of phenol.
