# Supplementary material for: Utilization of Carbon Nanospheres in Photocatalyst Production: From Composites to Highly Active Hollow Structures
Source: Materials (Basel). 2019 Aug 9;12(16):2537. doi: 10.3390/ma12162537 (PMC6720943; doi:10.3390/ma12162537)
Supplement: Supplementary file 1 [file materials-12-02537-s001.pdf]

materials

Article

Utilization of Carbon Nanospheres in Photocatalyst Production: from Composites to Highly Active Hollow Structures

Tamás Gyulavári [1,2], Gábor Veréb [1,3,*], Zsolt Pap [1,4,5,*], Balázs Réti [2], Kornelia Baan [2], Milica Todea [4,6], Klára Magyari [4], Imre Miklós Szilágyi [7] and Klara Hernadi [1,2]

1. Research Group of Environmental Chemistry, Institute of Chemistry, University of Szeged, H-6720 Szeged, Tisza Lajos krt. 103, Hungary
2. Department of Applied and Environmental Chemistry, University of Szeged, H-6720 Szeged, Rerrich tér 1, Hungary
3. Institute of Process Engineering, Faculty of Engineering, University of Szeged, H-6725 Szeged, Moszkvai krt. 9, Hungary
4. Nanostructured Materials and Bio-Nano-Interfaces Center, Interdisciplinary Research Institute on Bio-Nano-Sciences, Babes-Bolyai University, RO–400271 Cluj-Napoca, Treboniu Laurian 42, Romania
5. Institute of Environmental Science and Technology, University of Szeged, H-6720, Szeged, Tisza Lajos krt. 103, Hungary
6. Department of Molecular Sciences, Faculty of Medicine, Iuliu Haţieganu University of Medicine and Pharmacy, RO–400012 Cluj-Napoca, Romania
7. Department of Inorganic and Analytical Chemistry, Budapest University of Technology and Economics, Hungary

* Correspondence: verebg@mk.u-szeged.hu (G.V.); Tel.: +36-62-546-582 (G.V.); pzsolt@chem.u-szeged.hu (Z.P.); Tel.: +36-62-544-338 (Z.P.)

Supplementary material

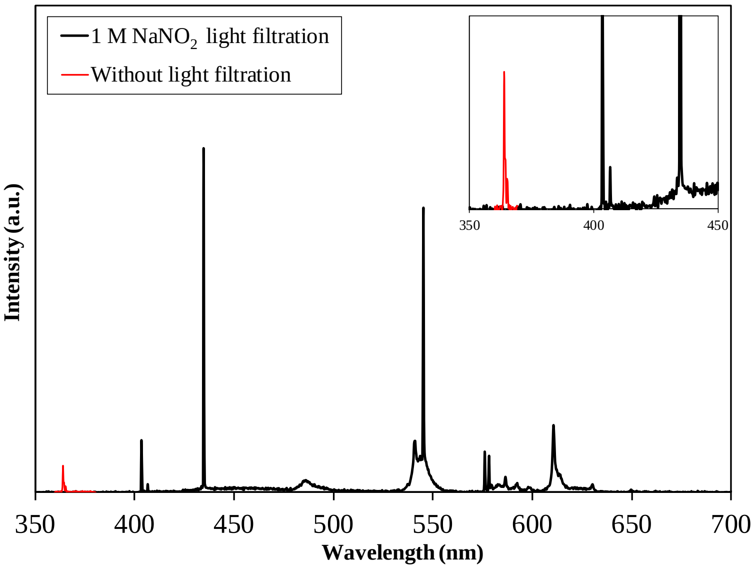

Figure S1. Emission spectrum of the visible light emitting lamps used for the photocatalytic activity measurements.

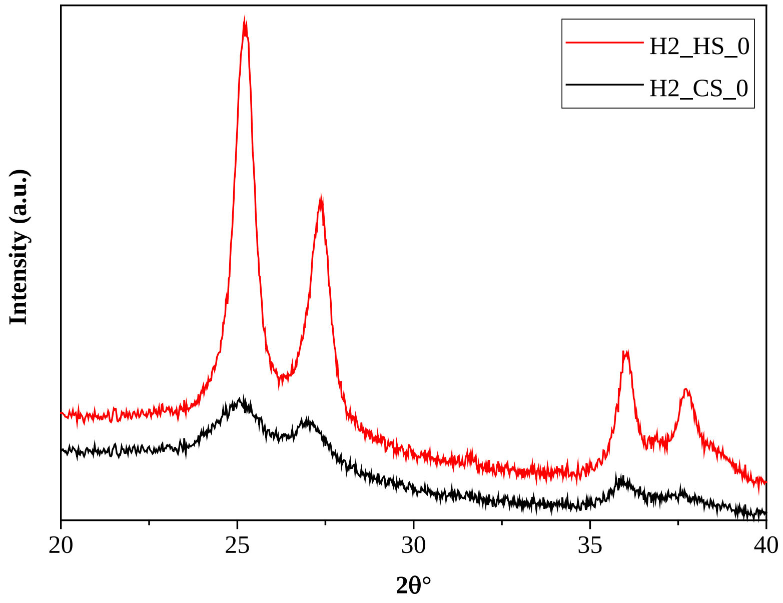

Figure S2. X-ray diffraction patterns of H2_CS_0 and H2_HS_0 samples.

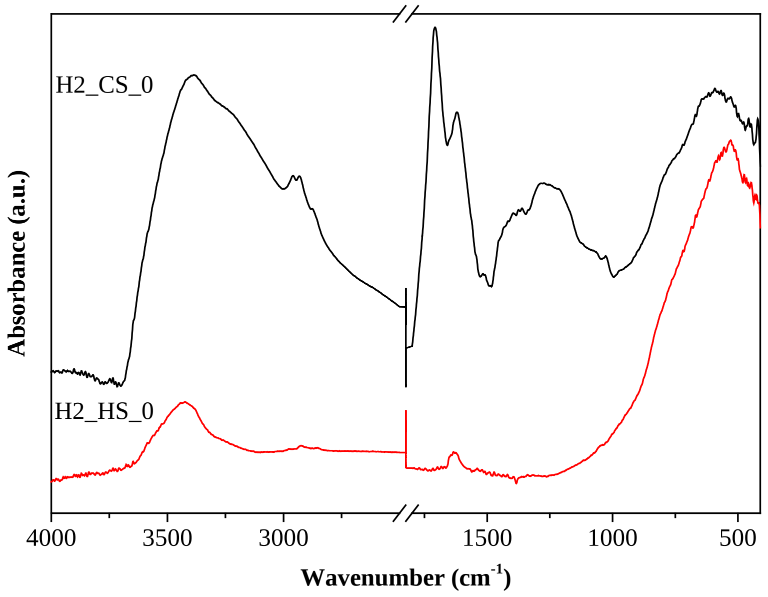

Figure S3. IR spectra of the investigated H2_CS_0 and H2_HS_0 samples.

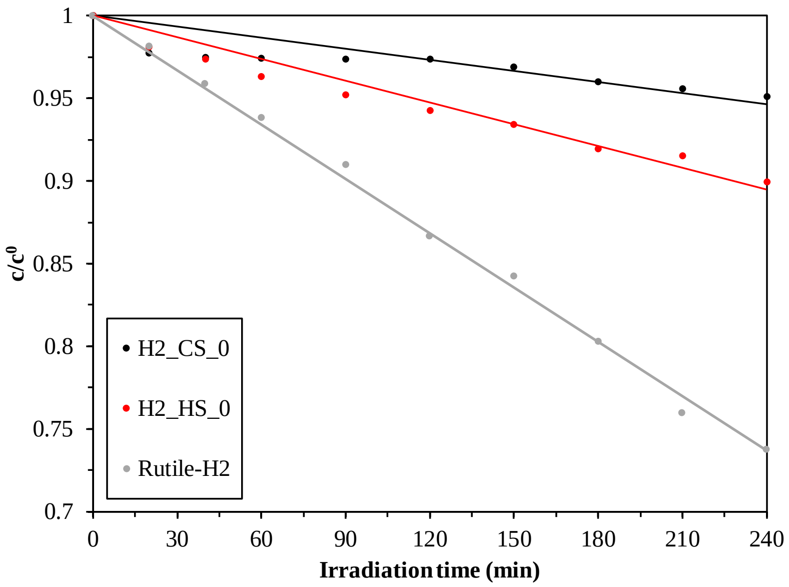

Figure S4. Phenol degradation curves of the H2_CS_0, H2_HS_0 and reference Rutile-H2 samples.

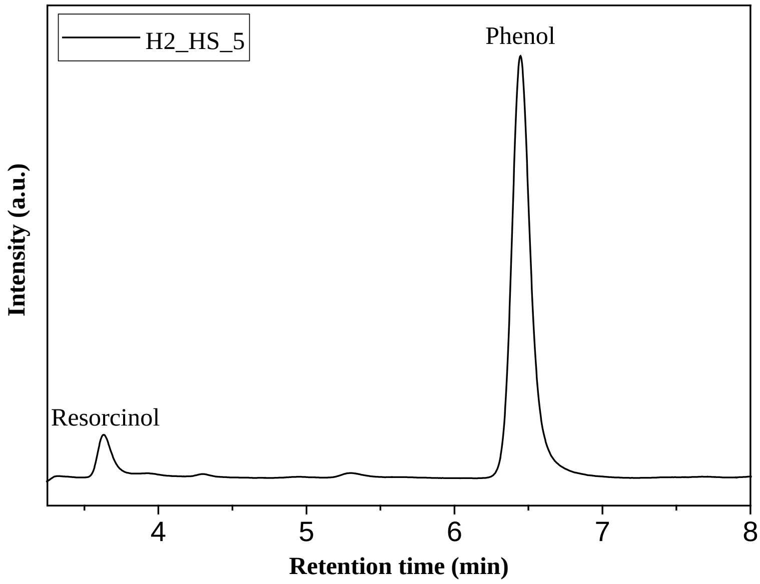

Figure S5. Chromatogram of the most efficient H2_HS_5 sample by the end of the photocatalytic oxidation of phenol.
